# FIBP is a prognostic biomarker and correlated with clinicalpathological characteristics and immune infiltrates in acute myeloid leukemia

**DOI:** 10.1007/s12672-023-00723-1

**Published:** 2023-06-13

**Authors:** Muya Ma, Lingling Xu, Wenhua Cui, Yan Huang, Gang Chi

**Affiliations:** 1 Department of Hematology, Changzhi People’s Hospital, The Affiliated Hospital of Changzhi Medical College, Changzhi, 046000 Shanxi China; 2 Department of Hematology, Yantai Yuhuangding Hospital, The Affiliated Hospital of Qingdao University, Shandong 264000 Yantai, China; 3 Department of Biochemistry, Changzhi Medical College, Changazhi, 046000 Shanxi China

**Keywords:** FIBP, Acute myeloid leukemia, Prognosis, Bioinformatic analysis, Immune infiltrates

## Abstract

Acute myeloid leukemia (AML) is one of the most common hematological malignancy that has a high recurrence rate. FIBP was reported to be highly expressed in multiple tumor types. However, its expression and role in acute myeloid leukemia remains largely unknown. The aim of this study was to clarify the role and value of FIBP in the diagnosis and prognosis, and to analyze its correlation with immune infiltration in acute myeloid leukemia by The Cancer Genome Atlas (TCGA) dataset. FIBP was highly expressed in AML samples compared to normal samples. The differentially expressed genes were identified between high and low expression of FIBP. The high FIBP expression group had poorer overall survival. FIBP was closely correlated with CD4, IL-10 and IL-2. The enrichment analysis indicated DEGs were mainly related to leukocyte migration, leukocyte cell–cell adhesion, myeloid leukocyte differentiation, endothelial cell proliferation and T cell tolerance induction. FIBP expression has significant correlation with infiltrating levels of various immune cells. FIBP could be a potential targeted therapy and prognostic biomarker associated with immune infiltrates for AML.

**Supplementary Information:**

The online version contains supplementary material available at 10.1007/s12672-023-00723-1.

## Introduction

Acute myeloid leukemia (AML) is the most common adult heterogeneous hematological malignancy that arises from clonal expansion of transformed hematopoietic stem and progenitor cells. It is associated with genomic alterations in cell proliferation and differentiation [1, 2]. It has a high incidence accounts for approximately 60% of all leukemia [3]. It seriously endangers human health and life. Chemotherapies are the main treatment for acute myeloid leukemia [3, 4]. However, there are still poor prognosis and short disease-free survival after chemotherapy. Therefore, it is urgent to find feasible molecular target for AML to complement existing therapeutic strategies.

FGF1 intracellular binding protein (FIBP) has been reported to be an intracellular protein and could bind to the acidic fibroblast growth factor (aFGF), which participated in cell proliferation by stimulating mitogenesis [5, 6]. FIBP might be involved in mitogenic activity and cell proliferation. The depletion of FIBP in breast cancer cells exhibited impaired proliferation and decreased cellular migration [7]. FIBP also increased tumorigenicity and induced chemotherapy resistance in colorectal cancer cells [8]. FIBP was highly expressed in tumors and a negative marker of antitumor T cells in solid tumors. FIBP KO enhanced T cell antitumor efficacy through downregulation of cholesterol metabolism [8, 9]. However, the role of FIBP in acute myeloid leukemia remains largely unknown.

Thus, we evaluated the prognostic value of FIBP expression in AML based on TCGA data. We investigated FIBP expression and its correlation with survival in AML patients to understand pathological process and aggressiveness in AML. We further investigated the hub genes and the important role of FIBP in the immune microenvironment through protein-protein interaction network and immune infiltration analysis. This study was expected to provide new targets for AML precise treatment and potential application in predicting AML prognosis.

## Materials and methods

### Data sources

The expression and clinical data of TCGA pan-cancer and GTEx data were downloaded from the UCSC Xena database [10] (https://xenabrowser.net/datapages/). AML clinical data were downloaded from TCGA database (https://portal.gdc.cancer.gov/). Patients with insufficient clinical information were not included. The RNA-Seq gene expression FPKM (Fragments Per Kilobase per Million) of 151 cases with AML and clinical data were retained and further analyzed. The HTSeq-FPKM data were transformed to TPM (transcription per million reads) for the following analysis. The healthy subjects and AML patient blasts used for ex vivo experiments were obtained from peripheral blood or bone marrow samples collected from Changzhi People’s Hospital, the Affiliated Hospital of Changzhi Medical College. The parents or guardians of each subject provided signed informed consent. The study protocol acquired approval from the ethics committee of Changzhi Medical College (No: RT2023001).

### Analysis of differentially expressed genes

The patients with AML were divided into high or low expression groups according to the median expression value of FIBP in TCGA samples. Expression profiles (level 3 HTSeq-Counts) were compared between high and low FIBP expression groups to identify differentially expressed genes (DEGs) using R Package DESeq2. |logFC|>1and FDR < 0.05 were considered as DEGs [11].

### Functional enrichment analysis

The Gene Ontology (GO) and Kyoto Encyclopedia of Genes and Genomes (KEGG) enrichment analyses were analyzed for DEGs using the ggplot2 package for visualization and the cluster Profiler package for statistical analysis [12].

### Diagnostic value analysis

The receiver operating characteristic (ROC) curve was used to assess the diagnostic value of FIBP in AML. The area value under the ROC curve is between 0.5 and 1. AUC in 0.5–0.7 has a low accuracy, AUC in 0.7–0.9 has a certain accuracy, and AUC above 0.9 has a high accuracy [13].

### Immune infiltration analysis by ssGSEA

The single sample gene set enrichment analysis (ssGSEA) method was performed using R package GSVA to analyze the immune infiltration of AML for 24 types of immune cells in tumor samples [14]. The relative enrichment score of each immunocyte was quantified from gene expression profile for each tumor sample based on the signature genes of the 24 types immunocyte. The correlation between FIBP and these immune cells was analyzed by Spearman correlation.

### Quantitative real-time PCR

The quantification of the expression of human genes was performed using real-time RT-PCR. The sequences of the primers used for detecting gene expression were as follows: *FIBP*, sense 5′-TGAGCTGGACATCTTCGTGG-3′, antisense 5′- GGTCACCGAGTAACCATCGAG-3′; *GAPDH*, sense 5′-TCGTCCCGTAGACAAA ATGG-3′, antisense 5′-TTGAGGTCA ATGAAGGGGTC-3′. For sample analysis, the threshold was set based on the exponential phase of products, and C_T_ value for samples was determined. The resulting data were analyzed with the comparative C_T_ method for relative gene expression quantification against GAPDH (house-keeping gene).

### Western blot analysis

Western blot assay was done as described previously [15]. Antibodies were purchased from ABclonal Technology (Wuhan, China). Briefly, 50 µg of protein was loaded on 10% SDS-Page gel. Following blotting, the blots were incubated with appropriate primary antibodies at 4 °C overnight. Later, the blots were incubated with appropriate HRP conjugated secondary antibodies at room temperature for an hour. ECL reagent was used for imaging the blots.

## Results

### FIBP expression analysis in pan-cancer and LAML

**Fig. 1:**
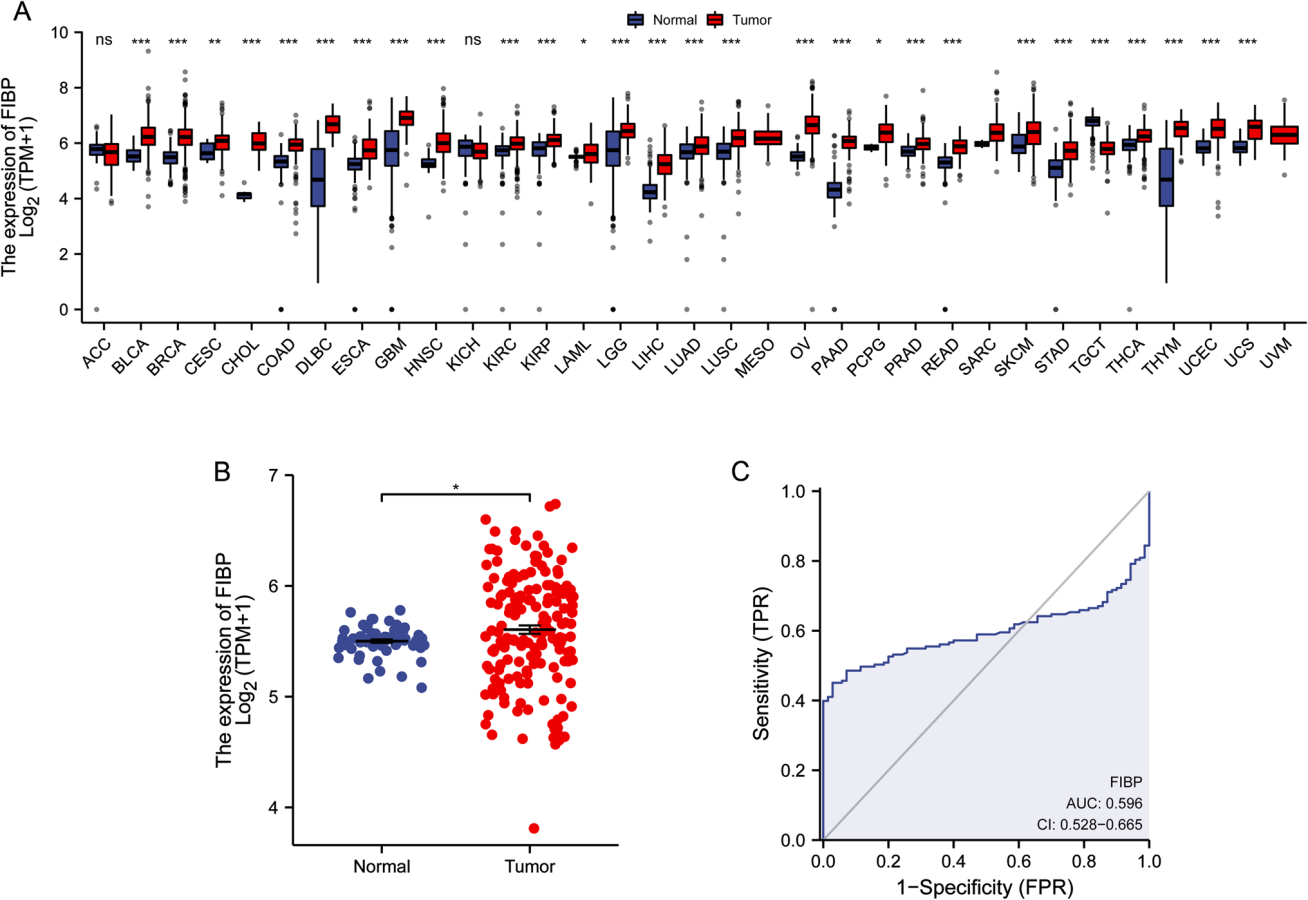
FIBP expression in pan-cancer and LAML.** A** FIBP expression between tumor tissues from TCGA and normal tissues from GTEx in pan-cancer. *P < 0.05, ***P < 0.001. **B** FIBP expression in GTEx normal samples and TCGA LAML samples. *P < 0.05. **C** ROC curve of FIBP. The area under the curve (AUC) values was considered as follows: AUC = 0.5 indicated noninformative; 0.5 < AUC ≤ 0.7 indicated low accurate; 0.7 < AUC ≤ 0.9 indicated moderately accurate; 0.9 < AUC < 1 indicated highly accurate; AUC = 1 indicated perfect

FIBP expression was explored in pan-cancer data from TCGA and GTEx. FIBP expression was significantly upregulated in 28 types of tumors than that in normal tissues, including BLCA, BRCA, CESC, CHOL, COAD, DLBC, ESCA, GBM, HNSC, KIRC, KIRP, LAML, LGG, LIHC, LUAD, LUSC, OV, PAAD, PCPG, PRAD, READ, SKCM, STAD, TGCT, THCA, THYM, UCEC, UCS (P < 0.05), while its expression was no significant difference between tumors and normal tissues including ACC, KICH, MESO, SARC and UVM (Fig. 1A). FIBP expression was further compared in 70 GTEx normal samples and 173 TCGA acute myeloid leukemia samples. FIBP was significantly upregulated in LAML samples (P < 0.05) (Fig. 1B). ROC analysis demonstrated that FIBP had a low diagnostic accuracy with AUC of 0.596 (Fig. 1C).

### Analysis of differentially expressed genes

**Fig. 2:**
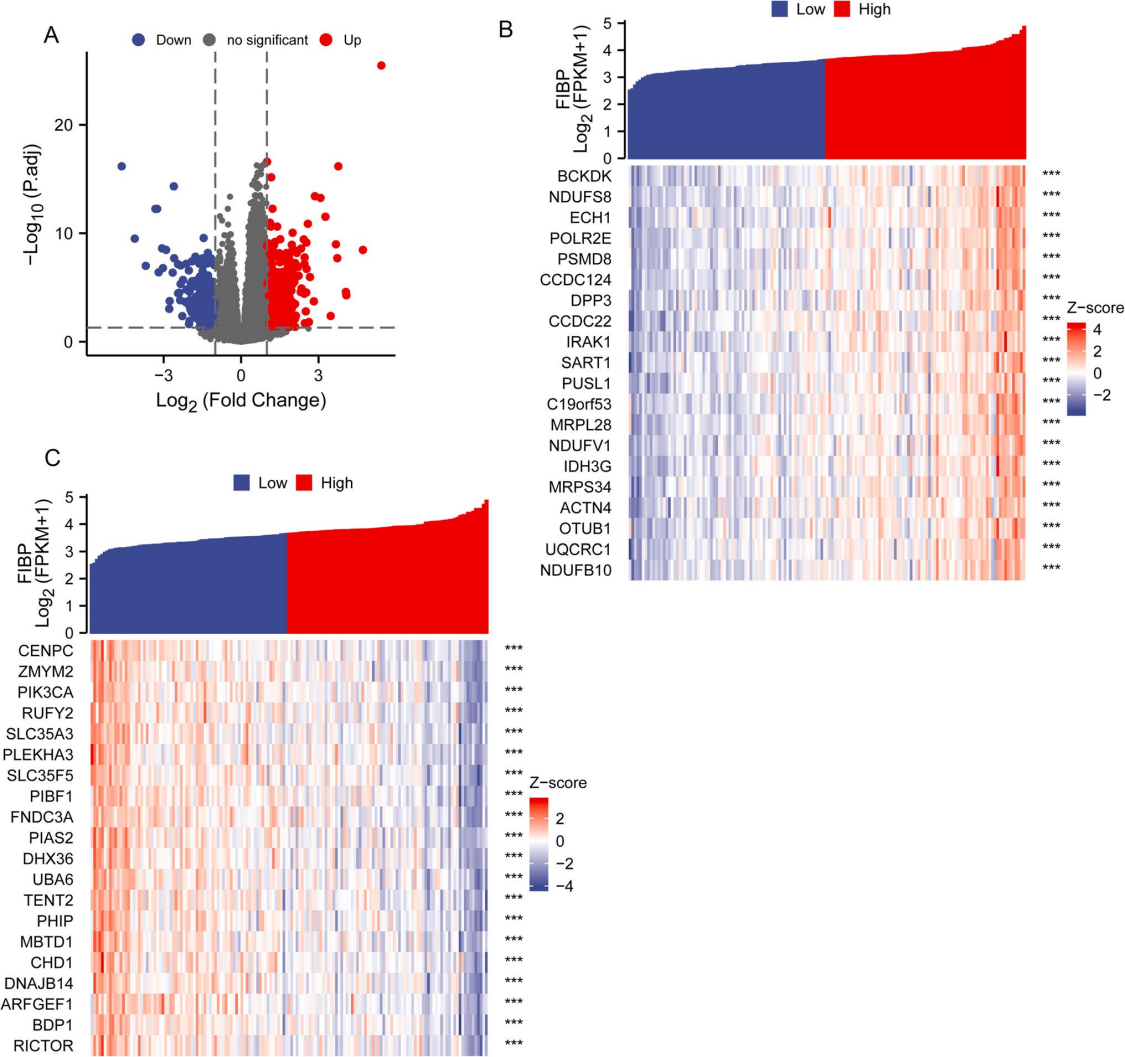
The differential gene expression map in the TCGA-LAML database.** A** The volcano plot of DEGs. Each point represents one gene; blue color indicated downregulation and red color indicated upregulation. **B** The heatmap of the top 20 differentially expressed genes in the high FIBP-expression LAML patients. The blue represent downregulated genes and the red represent upregulated gene. ***P < 0.001. **C** The heatmap of the top 20 differentially expressed genes in the low FIBP-expression LAML patients. Blue represents low expression, and red represents high expression. The blue represent downregulated genes and the red represent upregulated gene. ***P < 0.001

The differentially expressed genes (DEGs) were analyzed using TCGA cohort data and patients with LAML were divided into the high expression and the low expression group based on FIBP levels. A total of 720 differentially expressed genes were screened, including 411 upregulated genes and 309 downregulated genes (Fig. 2A). The gene expression heatmap was obtained for the top 20 differentially expressed genes in the high- and low FIBP-expression LAML patients (Fig. 2B, C).

### GO and KEGG enrichment analysis and PPI network

**Fig. 3:**
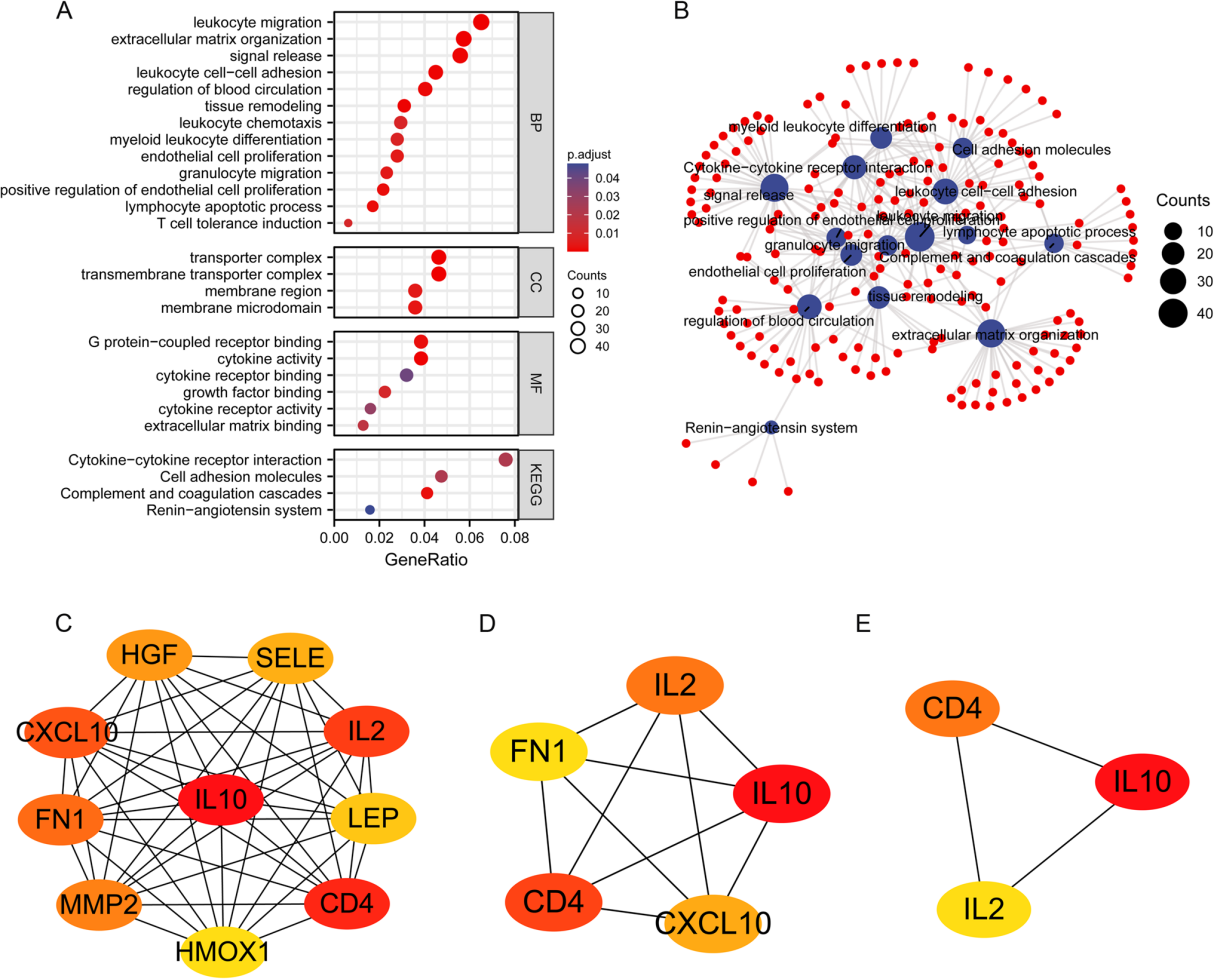
Protein–protein interaction (PPI) network and GO and KEGG analysis of DEGs between FIBP high and low expression groups in LAML.** A **and** B** GO and KEGG analysis of DEGs; **C–E **The hub genes of PPI network and MCODE2 components identified

The GO and KEGG enrichment analysis of DEGs were conducted and the primary BP contained leukocyte migration, extracellular matrix organization, signal release, leukocyte cell-cell adhesion, regulation of blood circulation, tissue remodeling, leukocyte chemotaxis, myeloid leukocyte differentiation, endothelial cell proliferation, granulocyte migration, positive regulation of endothelial cell proliferation, lymphocyte apoptotic process and T cell tolerance induction. The CC was mainly enriched in transporter complex, transmembrane transporter complex, membrane region and membrane microdomain. The MF was primarily involved in G protein-coupled receptor binding, cytokine activity, cytokine receptor binding, growth factor binding, cytokine receptor activity and extracellular matrix binding. The KEGG pathway enrichment was mainly related to cytokine-cytokine receptor interaction, cell adhesion molecules, complement and coagulation cascades and renin-angiotensin system (Fig. 3A, B). Furthermore, the top 10 hub genes of 720 DEGs were identified including HGF, SELE, IL-2, LEP, CD4, HMOX1, MMP2, FN1, CXCL10 and IL-10 (Fig. 3C). The top 5 hub genes were IL-2, IL-10, CXCL10, CD4 and FN1 among them (Fig. 3D). The top 3 hub genes were CD4, IL-10 and IL-2 (Fig. 3E). The relationship between FIBP and the top 10 genes was also explored and the result showed that FIBP had a significant positive correlation with CD4, CXCL10 and HMOX1, whereas FIBP was a significantly negatively correlated with HGF, LEP and SELE. However, no significant correlation was found between FIBP and FN1, IL2, IL10 and MMP2 (Supplementary Fig. S1A). Compared with the normal group, the expression level of HGF, CD4, HMOX1, MMP2 and IL-10 was significantly increased in AML group, whereas the expression level of LEP and FN1 was significantly decreased. There was no significant difference in IL-2 and CXCL10 expression between AML and normal group (Supplementary Fig. S1B).

### Association between FIBP expression and clinicopathological characteristics

**Fig. 4:**
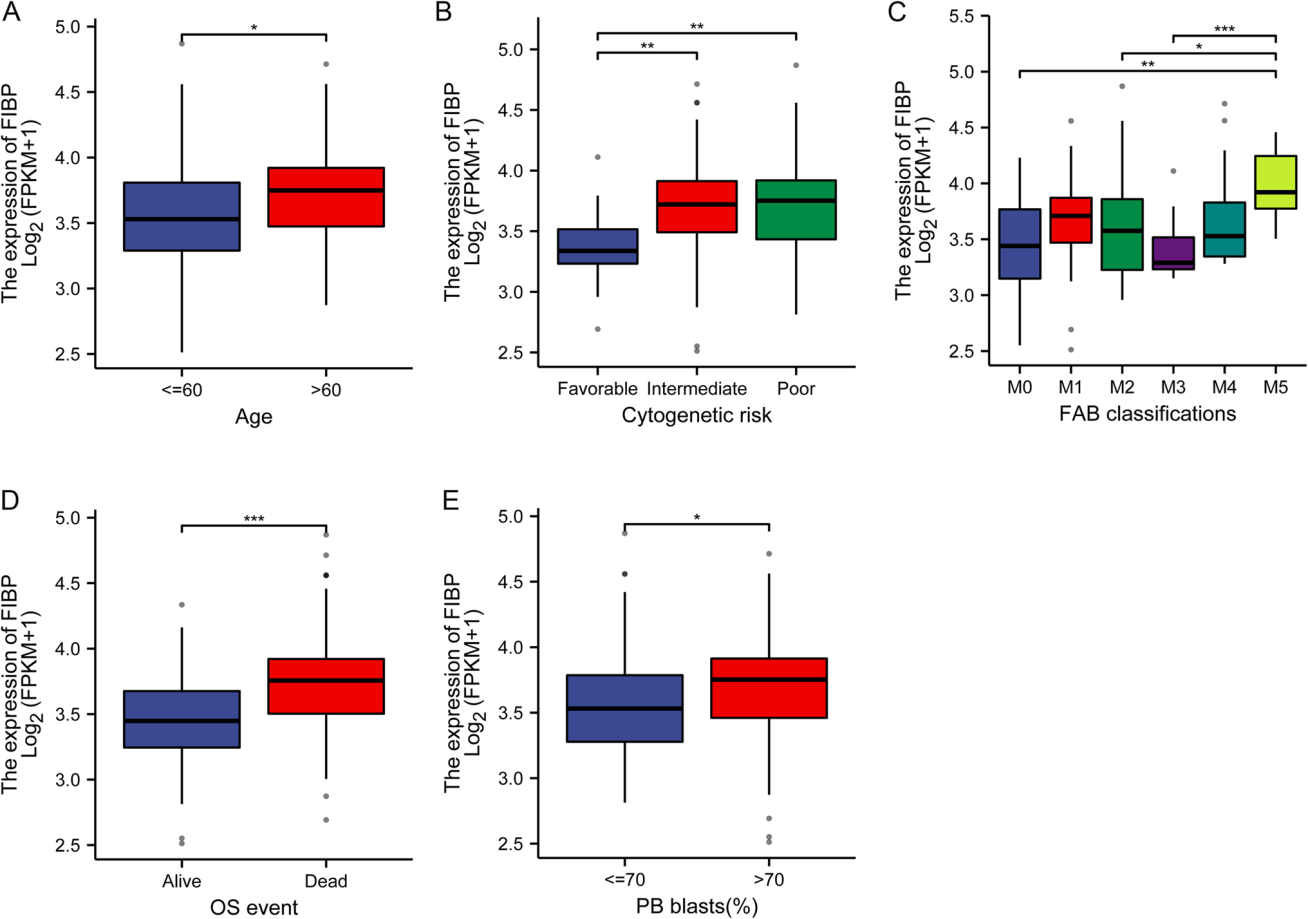
Associations between the FIBP expression and clinicopathological characteristics.** A** age (≤ 60 and > 60), **B** cytogenetic risk (Favorable, Intermediate and Poor), **C** FAB classifications (M0, M1, M2, M3, M4 and M5), **D** OS event (Alive and Dead), **E** PB blasts (≤ 70% and > 70%). *P < 0.05, **P < 0.01, ***P < 0.001

Correlation analysis revealed that FIBP expression was significantly associated with WBC count (p < 0.05), PB blasts (p < 0.01), FAB classifications (p < 0.01) and Cytogenetic risk (p < 0.001). No correlation was found between FIBP expression and other clinicopathologic characteristics (Table 1). Univariate logistic regression analysis revealed that FIBP upregulation in LAML was significantly associated with WBC count (p < 0.05), PB blasts (p < 0.01), Cytogenetic risk (p < 0.001), and NPM1 mutation (p < 0.05) (Table 2). The higher FIBP expression was significantly correlated with age (p < 0.05), cytogenetic risk (Favorable vs. Intermediate, Favorable vs. Poor, p < 0.01), FAB classifications (M0 vs. M5, p < 0.01; M2 vs. M5, p < 0.05; M3 vs. M5, p < 0.001), OS event (p < 0.001) and PB blasts (p < 0.05) (Fig. 4).

**Table 1:** Association between FIBP expression and clinicopathological characteristics in AML patients

Characteristic	Levels	Low expression of FIBP	High expression of FIBP	p
n		75	76	
Gender, n (%)	Female	36 (23.8%)	32 (21.2%)	0.573
	Male	39 (25.8%)	44 (29.1%)	
Age, n (%)	< = 60	49 (32.5%)	39 (25.8%)	0.114
	> 60	26 (17.2%)	37 (24.5%)	
WBC count (x10^9/L), n (%)	< = 20	45 (30%)	32 (21.3%)	0.033
	> 20	29 (19.3%)	44 (29.3%)	
BM blasts(%), n (%)	< = 20	34 (22.5%)	26 (17.2%)	0.219
	> 20	41 (27.2%)	50 (33.1%)	
PB blasts(%), n (%)	< = 70	45 (29.8%)	27 (17.9%)	0.004
	> 70	30 (19.9%)	49 (32.5%)	
FAB classifications, n (%)	M0	10 (6.7%)	5 (3.3%)	0.001
	M1	14 (9.3%)	21 (14%)	
	M2	21 (14%)	17 (11.3%)	
	M3	12 (8%)	3 (2%)	
	M4	17 (11.3%)	12 (8%)	
	M5	1 (0.7%)	14 (9.3%)	
	M6	0 (0%)	2 (1.3%)	
	M7	0 (0%)	1 (0.7%)	
FLT3 mutation, n (%)	Negative	55 (37.4%)	47 (32%)	0.104
	Positive	17 (11.6%)	28 (19%)	
RAS mutation, n (%)	Negative	69 (46%)	73 (48.7%)	0.491
	Positive	5 (3.3%)	3 (2%)	
Cytogenetic risk, n (%)	Favorable	25 (16.8%)	6 (4%)	< 0.001
	Intermediate	36 (24.2%)	46 (30.9%)	
	Poor	14 (9.4%)	22 (14.8%)	

**Table 2:** Univariate logistic regression analysis between FIBP expression and clinical pathological characteristics

Characteristics	Total(N)	Odds Ratio(OR)	P value
Gender (Male vs. Female)	151	1.269 (0.668–2.421)	0.467
Race (White vs. Asian&Black or African American)	149	1.436 (0.474–4.572)	0.523
Age (> 60 vs. < = 60)	151	1.788 (0.933–3.466)	0.082
WBC count (x10^9/L) (> 20 vs. < = 20)	150	2.134 (1.117–4.129)	0.023
BM blasts (%) (> 20 vs. < = 20)	151	1.595 (0.829–3.095)	0.164
PB blasts (%) (> 70 vs. < = 70)	151	2.722 (1.419–5.316)	0.003
Cytogenetic risk (Intermediate and Poor vs. Favorable)	149	5.667 (2.290–16.189)	< 0.001
FLT3 mutation (Positive vs. Negative)	147	1.927 (0.948–4.006)	0.073
IDH1 R132 mutation (Positive vs. Negative)	149	0.589 (0.171–1.857)	0.374
IDH1 R140 mutation (Positive vs. Negative)	149	0.684 (0.194–2.245)	0.533
RAS mutation (Positive vs. Negative)	150	0.567 (0.113–2.399)	0.449
NPM1 mutation (Positive vs. Negative)	150	2.333 (1.057–5.406)	0.040

### Prognostic value of FIBP in LAML

**Fig. 5:**
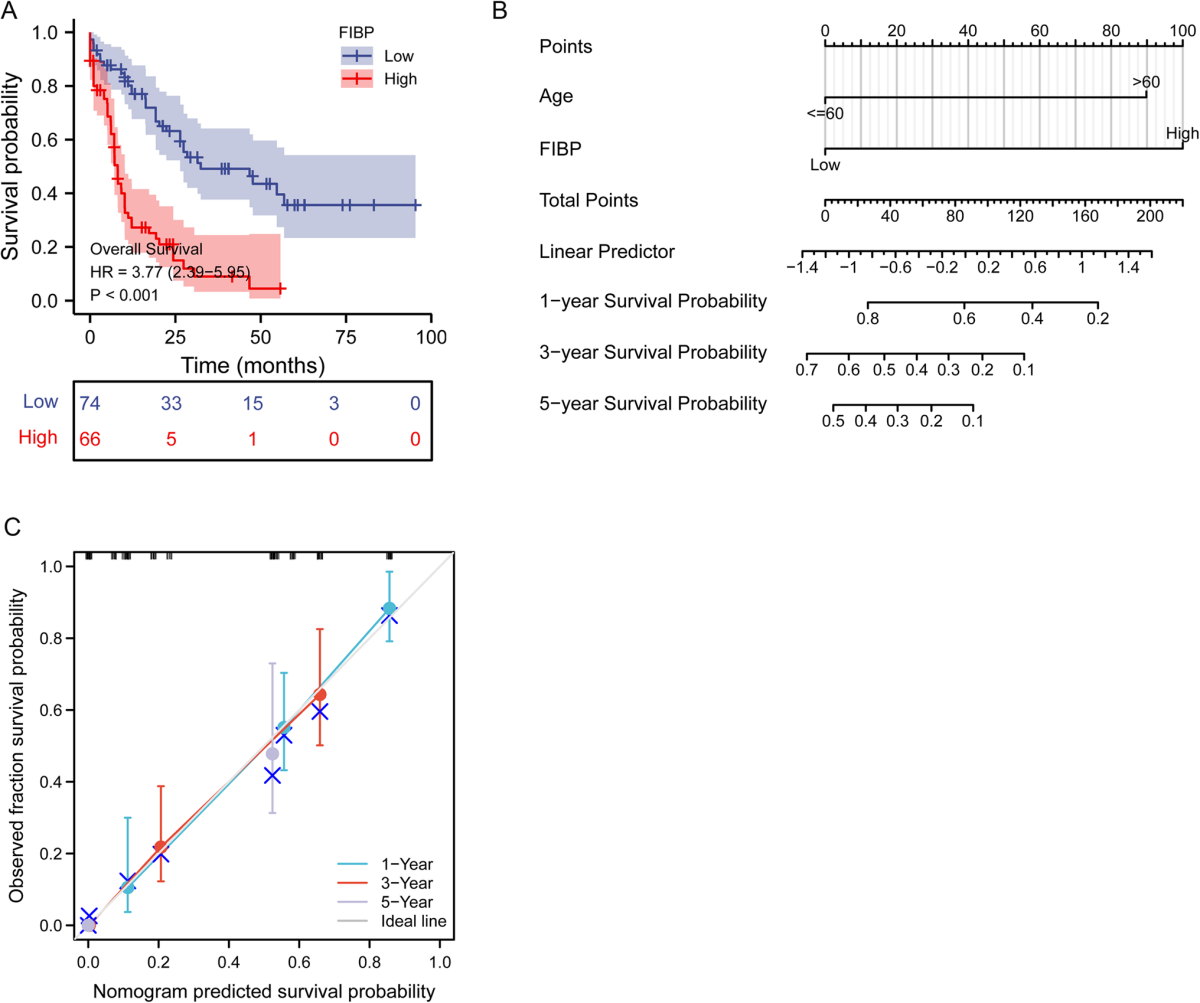
Analysis of prognostic value of FIBP in LAML. **A** Overall survival curve of LAML patients with high and low FIBP expression levels. HR: hazard ratio. **B** Nomogram for predicting the probability of 1-, 3-, and 5-year OS for LAML patients. **C** Calibration plot of the nomogram for predicting the probability of OS at 1, 3, and 5 years

To confirm the correlation between FIBP expression and LAML prognosis, survival rates were compared between the high and low FIBP level groups. The Kaplan–Meier survival analysis indicated that the LAML patients with high FIBP expression had poorer overall survival (HR = 3.77(2.39–5.95), p < 0.001) (Fig. 5A). Multivariate analyses showed that FIBP remained independently associated with overall survival (HR = 3.571(2.191–5.821), p < 0.001), along with age (p < 0.001) in Table 3. The age and FIBP expression were included in the nomogram based on Cox proportional hazards regression model (Fig. 5B). The calibration plots were constructed to evaluate the agreement between predicted and actual OS for the prognosis model, and the results showed that the predicted results of the nomogram were reliable (Fig. 5C).

**Table 3:** Univariate and multivariate analyses of overall survival in AML patients

Characteristics	Univariate analysis		Multivariate analysis	
	HR (95% CI)	P value	HR (95% CI)	P value
Gender (Female vs. Male)	1.030 (0.674–1.572)	0.892		
Age ( < = 60 vs. >60)	3.333 (2.164–5.134)	**< 0.001**	3.298 (2.093–5.196)	**< 0.001**
Cytogenetic risk (Favorable vs. Intermediate and Poor)	3.209 (1.650–6.242)	**< 0.001**	1.708 (0.844–3.457)	0.136
WBC count(x10^9/L) ( < = 20 vs. >20)	1.161 (0.760–1.772)	0.490		
NPM1 mutation (Negative vs. Positive)	1.137 (0.706–1.832)	0.596		
FIBP (Low vs. High)	3.772 (2.393–5.945)	**< 0.001**	3.571 (2.191–5.821)	**< 0.001**

### Relationship between FIBP expression and tumor-infiltrating immune cells

**Fig. 6:**
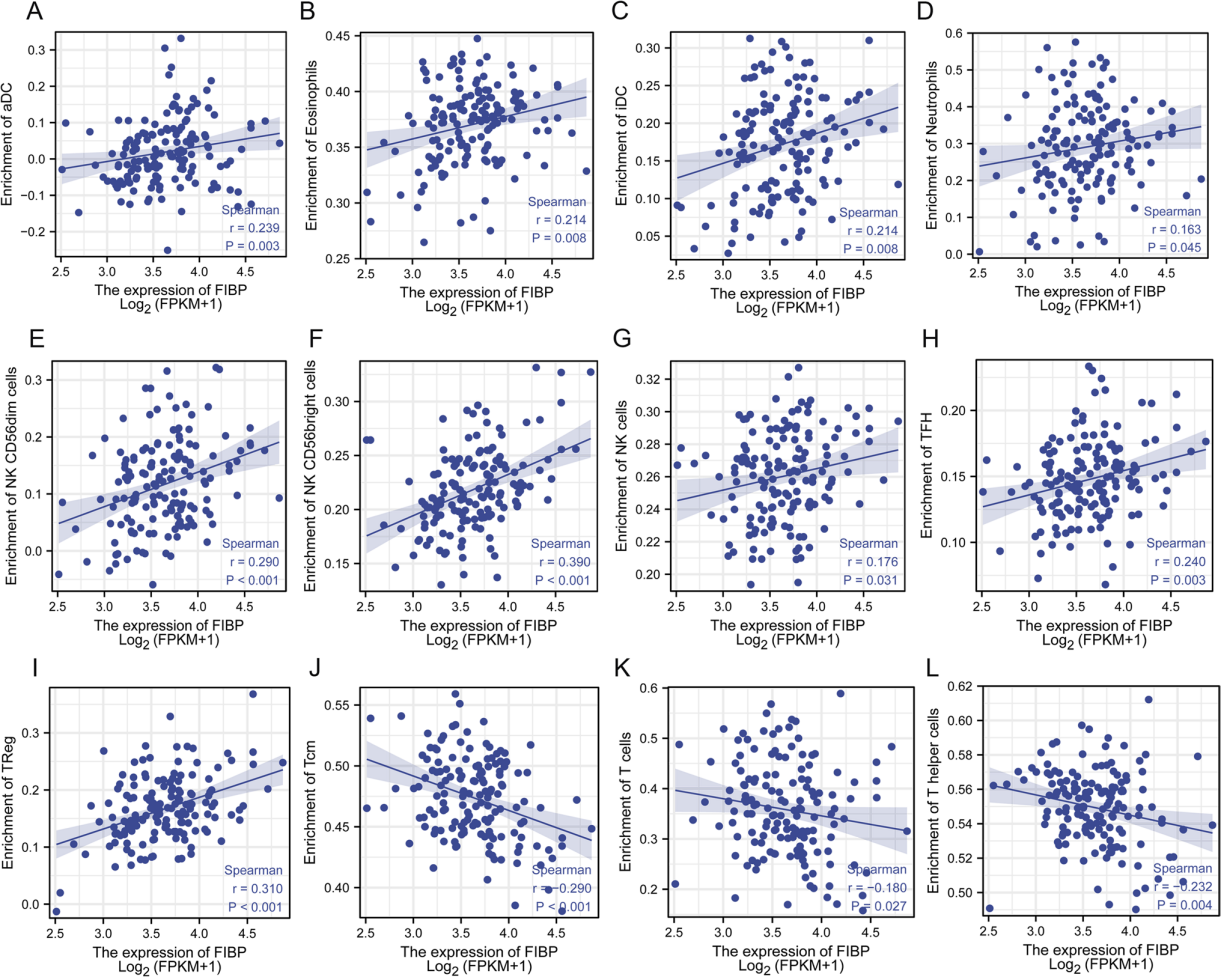
Relationship between FIBP expression and tumor-infiltrating immune cells.** A** aDC, **B** Eosinophils, **C** iDC, **D** Neutrophils, **E** NK CD56dim cells, **F** NK CD56bright cells, **G** NK cells, **H** TFH, **I** Treg, **J** Tcm, **K **T cells and **L** T helper cells. r: spearman’s correlation coefficient, r < 0 was considered as a negative correlation, and r > 0 was considered a positive correlation. P < 0.05 means statistically significant

To confirm whether FIBP expression was associated with tumor-infiltrating immune cells in LAML, Spearman correlation was performed to show the association between the expression of FIBP and the GSVA enrichment scores of immune cell infiltration calculated from RNA-seq in LAML tumor microenvironment. FIBP was positively correlated with aDC, eosinophils, iDC, neutrophils, NK CD56dim cells, NK CD56birght cells, NK cells, TFH and Treg (Fig. 6A–I), whereas it was negatively correlated with Tcm, T cells and T helper cells (Fig. 6J–L).

### Expression validation for FIBP gene in human acute myeloid leukemia

**Fig. 7:**
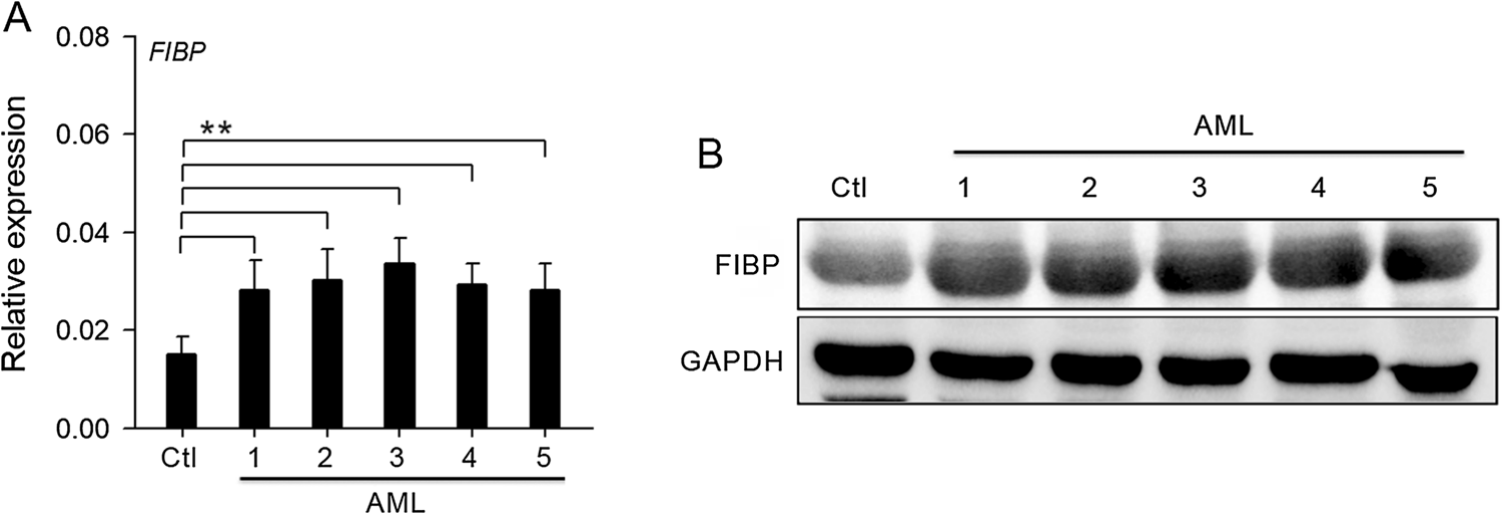
Expression validation for FIBP gene in acute myeloid leukemia patients. qPCR (**A**) and Western blot analysis (**B**) of FIBP were performed in bone marrow samples from AML patients and healthy volunteers. GAPDH was used as a normalizer. Ctl: healthy volunteers. **P < 0.01

To further investigate FIBP expression in AML patients, qPCR and Western blot were performed and showed FIBP high expression in AML patients compared with the healthy control (Fig. 7A, B and Supplementary  Fig.S2).

## Discussion

FIBP was an intracellular protein binding selectively to acidic fibroblast growth factor (aFGF), which regulated cell proliferation for multiple cell types by stimulating mitogenesis or inducing morphological changes [6, 16]. Studies have shown that FIBP increased tumorigenicity and was highly expressed in colon carcinoma [17]. FIBP knockdown increased sensitization of chemoresistant cells and attenuated cancer stemness [9, 18]. Moreover, it was showed that FIBP was also highly expressed in skin carcinogenesis and was involved in tumor cell cycle processes by regulating the key downstream target cyclin D1 [19]. To date, the role of FIBP in acute myeloid leukemia has not been investigated.

In this study, bioinformatics analysis based on TCGA data demonstrated that the expression of FIBP was significantly higher in AML samples than normal samples, indicating that FIBP played a role in tumorigenesis and progression. In addition, ROC analysis showed that FIBP might be a potential diagnostic biomarker. The relationship between FIBP expression and clinicopathological factors was further explored, and high FIBP protein expression was significantly associated with age (p < 0.05), cytogenetic risk (p < 0.01), FAB classifications (p < 0.001), OS event (p < 0.001) and PB blasts (p < 0.05). Kaplan–Meier survival analysis indicated that the high expression of FIBP was correlated with poorer overall survival times. Multivariate Cox regression analysis showed that FIBP was an independent prognostic factor affecting survival of AML patients (P < 0.001).

To explore the biological functions of FIBP, DEGs were analyzed based on AML patients with high or low FIBP expression from TCGA data. A total of 720 differentially expressed genes were identified and the functional enrichment analysis of these DEGs was performed in AML samples. The results demonstrated that these DEGs were mainly enriched in BP terms associated with leukocyte migration, extracellular matrix organization, signal release, leukocyte cell–cell adhesion, regulation of blood circulation, tissue remodeling, leukocyte chemotaxis, myeloid leukocyte differentiation, endothelial cell proliferation, granulocyte migration, positive regulation of endothelial cell proliferation, lymphocyte apoptotic process and T cell tolerance induction. MF was primarily involved in G protein-coupled receptor binding, cytokine activity, cytokine receptor binding, growth factor binding, cytokine receptor activity and extracellular matrix binding. It has been reported that Interactions between AML blasts and their adjacent endothelial cells in the bone marrow microenvironment were important for chemotherapy sensitivity [20]. AML cells have been confirmed to secrete angioregulatory mediators for stimulating endothelial cell proliferation and inducing angiogenesis [21, 22]. Moreover, the chemotherapy-resistant leukemic cells were surrounded by stromal cells, which promote AML cells survival by enabling them to evade immune destruction [23]. Therefore, FIBP may be essential for promoting AML proliferation and angiogenesis by these biological processes and pathways.

AML is highly dependent on the immune microenvironment for survival and growth [24, 25]. Therefore, the difference in immune cell infiltration between patients with high and low FIBP expression was compared in this study. FIBP was negatively correlated with Tcm (R = − 0.290, p < 0.001), T cells (R = − 0.180, p = 0.027) and T helper cells (R = − 0.232, p = 0.004), while it was positively correlated with aDC (R = 0.239, p = 0.003), Eosinophils (R = 0.214, p = 0.008), iDC (R = 0.214, p = 0.008), Neutrophils (R = 0.163, p = 0.045), NK CD56dim cells (R = 0.290, p < 0.001), NK CD56 bright cells (R = 0.390, p < 0.001), NK cells (R = 0.176, p = 0.031), TFH (R = 0.240, p = 0.003) and Treg (R = 0.310, p < 0.001). Multiple clinical studies have demonstrated various disruptions of T cell immunity in AML. T cell numbers and functions are altered to favor the progression of acute myeloid leukemia. A higher frequency of Tregs could impair the cell-mediated anti-leukemia immune response and was considered as a pivotal regulator of immune escape [26–29]. FIBP high expression may inhibit T cells and T helper cells numbers and increase the frequency of Treg cells to promote AML development. FIBP knockout consistently promoted T cell-mediated cancer killing and significantly reduced tumor size [8]. On the other hand, it has been reported that AML was also capable of inhibiting NK cell maturation and effector function and the loss of peripheral CD56 bright NK cells were found in AML patients [30, 31]. Importantly, NK cells are a type of innate lymphoid cell (ILC) and AML microenvironment creates the possibility of disrupting this balance of ILCs to drive the development of other ILC subsets at the expense of cytotoxic NK cells [32]. Thus, FIBP high expression was positively correlated with NK cells, but FIBP expression possibly increased NK cells with developmental defects.

In conclusion, these findings in this study determined FIBP may be a potential poor prognostic biomarker, which could aid clinicians in clinical application, assessment and therapeutics for AML. Future researches are required to include experiments in vivo and in vitro and enroll more patients to further verify these conclusions.

## Supplementary Information

Supplementary material 1

## Data Availability

All relevant data are within the paper and TCGA database: https://portal.gdc.cancer.gov/.
