# Supplementary material for: FIBP is a prognostic biomarker and correlated with clinicalpathological characteristics and immune infiltrates in acute myeloid leukemia
Source: Discov Oncol. 2023 Jun 13;14:97. doi: 10.1007/s12672-023-00723-1 (PMC10264308; doi:10.1007/s12672-023-00723-1)
Supplement: Supplementary file 1 — Supplementary material 1 [file 12672_2023_723_MOESM1_ESM.doc]

**FIBP is a prognostic biomarker and correlated with clinicalpathological characteristics and immune infiltrates in acute myeloid leukemia**


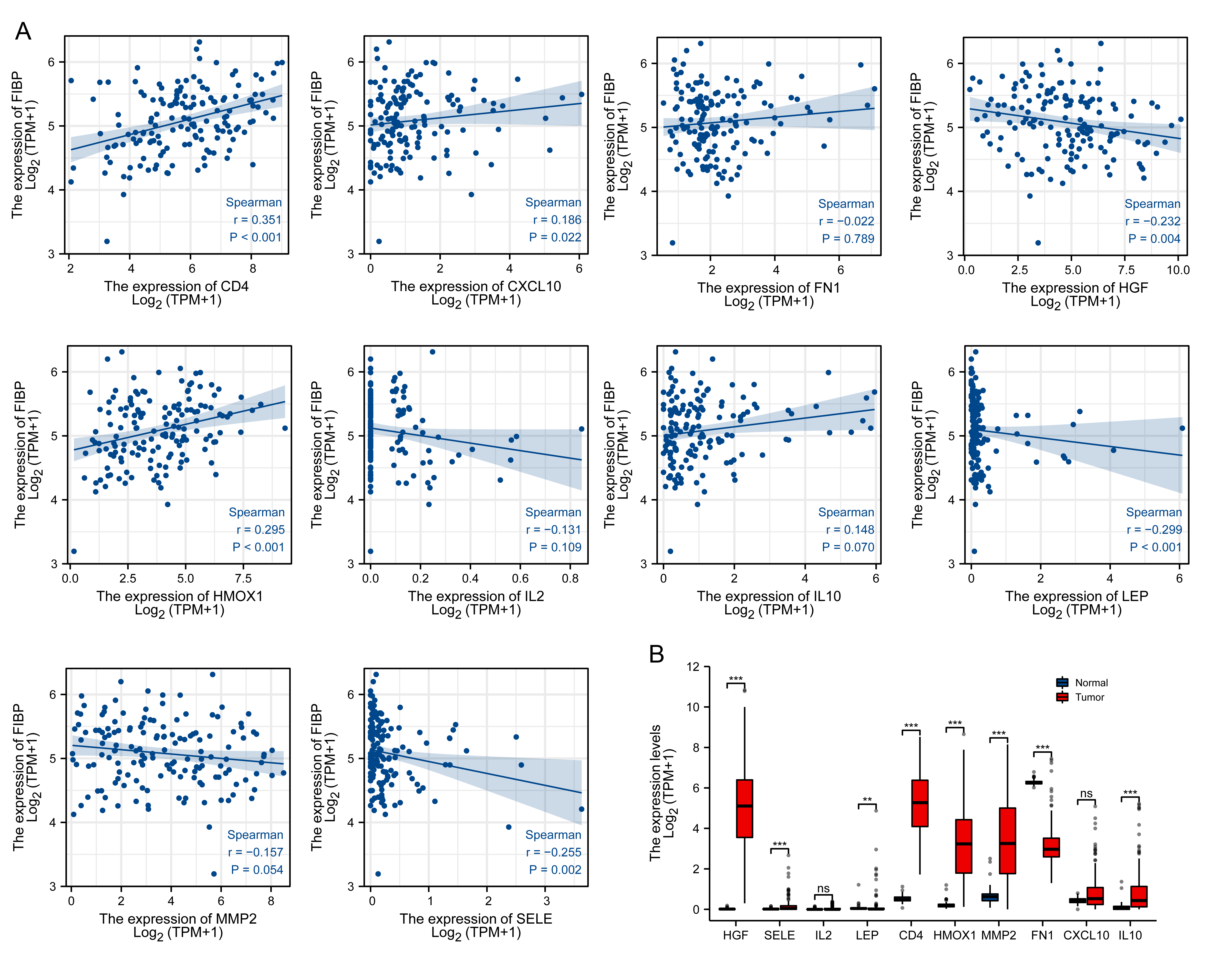


**Supplementary Figure S1: The expression of the top 10 genes and the relationship between these genes and FIBP in AML. (A)**. Analysis of the correlation between FIBP and the top 10 genes. r: spearman’s correlation coefficient, r＜0 was considered as a negative correlation, and r＞0 was considered a positive correlation. **(B)**. The RNA expression level of the top 10 genes between AML from TCGA database and normal samples from the GTEx database. **P<0.01, ***P<0.001.


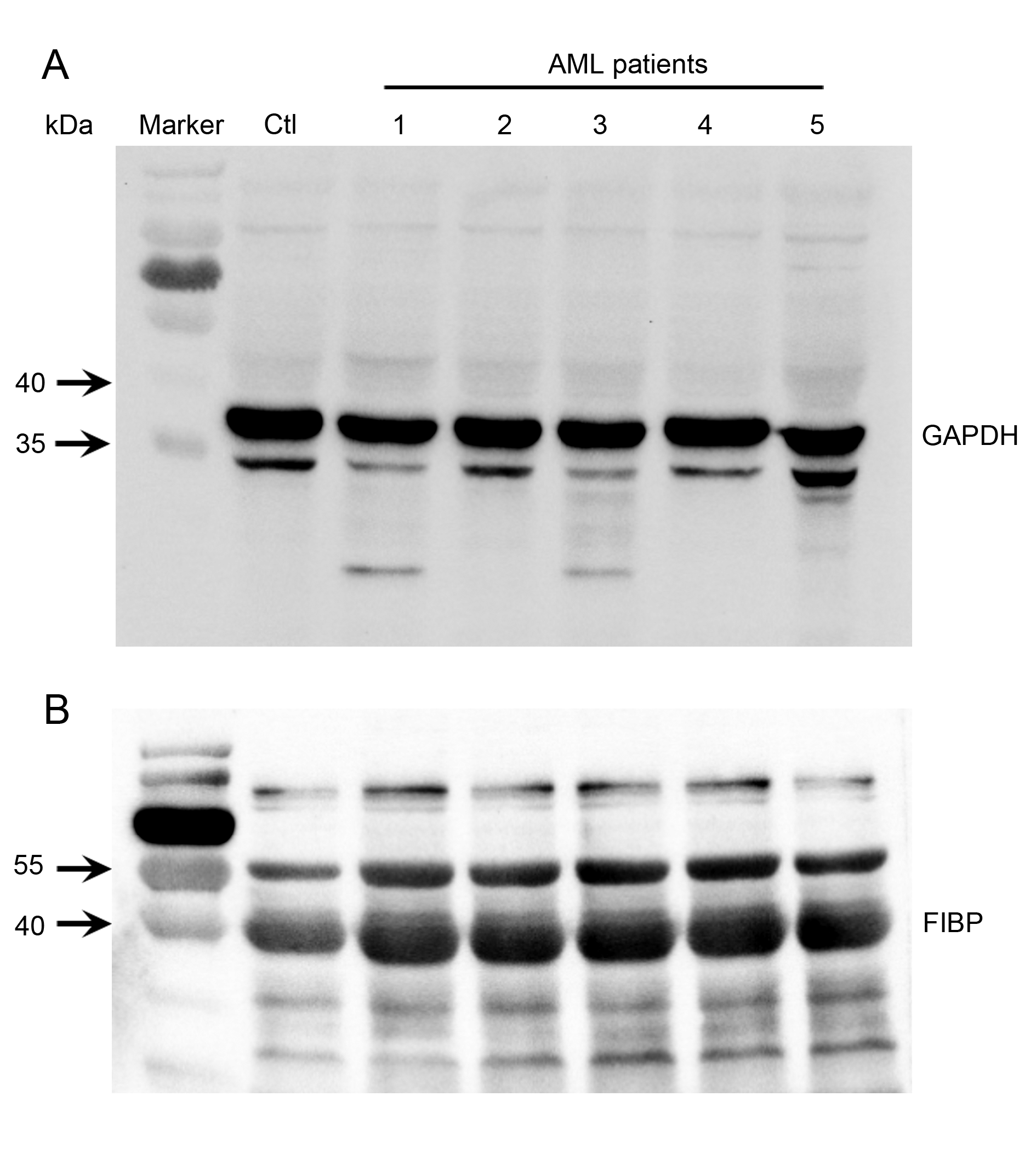


**Supplementary Figure S2:** Western blot was used to detect the protein expression level of FIBP in bone marrow samples from AML patients and healthy volunteers. **(A)** GAPDH expression was shown. **(B)** FIBP expression was shown. Ctl: healthy volunteers.
